# Determination of pyrethroid residues in herbal tea using temperature-controlled ionic liquid dispersive liquid-liquid microextraction by high performance liquid chromatography

**DOI:** 10.1038/s41598-020-61755-z

**Published:** 2020-03-13

**Authors:** Rui Zhang, Zhenchao Tan, Junlong Zhao, Yan Wen, Shuai Fan, Chenglan Liu

**Affiliations:** Key Laboratory of Natural Pesticide and Chemical Biology, Ministry of Agriculture & Key Laboratory of Bio-Pesticide Innovation and Application of Guangdong Province, South China Agricultural University, 483 Wushan Road, Guangzhou, 510642 China

**Keywords:** Environmental sciences, Risk factors, Chemistry

## Abstract

A simple and effective method for determining five pyrethroid residues in herbal tea by ultrasound-enhanced temperature-controlled (UETC) ionic liquid dispersive liquid-liquid microextraction (IL-DLLME) coupled with high performance liquid chromatography-diode array detection (HPLC-DAD) was developed. The use of ultrasonication and heating improved the ability of the ionic liquid to extract the analytes. Various parameters that affect the extraction efficiency were investigated and optimized using single factor experiments and response surface design. The optimum conditions of the experiment were 121 µL of [HMIM][PF6] (extraction solvent), 794 µL of acetonitrile (dispersive solvent), a heating temperature of 40°C, a sonication time of 3.6 min and a pH of 2.9. Under optimized conditions, the linearity was in the range of 0.05–5 mg L^−1^ with correlation coefficients above 0.9993. The limits of detection and quantification were 1.25–1.35 µg L^−1^ and 5 µg L^−1^, respectively. The mean recoveries of the five pyrethroids ranged from 74.02% to 109.01%, with RSDs below 9.04%. The proposed method was reliable for the analysis of pyrethroids in Chinese herbal tea.

## Introduction

Herbal tea is a kind of herbal beverage made from Chinese herbal medicine materials through a series of processing steps that has many medical functions, such as detoxification, thirst stimulation, and disease control. Herbal tea is a very popular drink in southern China and was recognized as a national intangible cultural heritage in 2006^1^. Pesticides are essential for controlling pests and diseases in Chinese herbal medicines. There are many reports about the detection of pesticide residues in herbal plants^2–8^. At present, synthetic pyrethroid insecticides, which have similar structures to that of natural pyrethrum found in the chrysanthemum species, are commonly used worldwide because of their high insecticidal activity, low persistence and relatively low toxicity to birds and mammals^9,10^. Studies have shown that pyrethroid pesticides are toxic to the nervous, reproductive, immune and cardiovascular systems^11^. The widespread application of pyrethroids during herbal plant cultivation is one of the main sources of these residues in herbal tea. Nevertheless, pyrethroid residues in herbal tea pose a potential threat to consumers. Consequently, it is urgent to develop a simple and sensitive analytical method for the detection of the pyrethroid residues in herbal tea.

Various methods have been established for the determination of pyrethroid residues in several types of samples^10,12–17^. Some pretreatment procedures, such as liquid-liquid extraction (LLE), solid-phase extraction (SPE), and dispersive solid phase extraction (DSPE), were used for the extraction and clean-up of pyrethroid in various samples. Although many of these extraction approaches are suitable and effective, there are also some disadvantages, including tedious procedures, expensive costs, a large organic solvent usage and a high consumption of time. In recent years, a novel liquid-phase microextraction method, denoted as dispersive liquid-liquid microextraction (DLLME), has been developed^18^. The traditional DLLME process consists of a ternary component solvent system (a dispersive solvent, an extraction solvent, and an aqueous sample). The method has some merits, including simplicity, efficiency, inexpensive cost, and high enrichment factors^19,20^. Recently, a variety of modifications to DLLME have been reported, such as *in-situ* derivatization DLLME^21,22^, and green extractants for DLLME^23,24^. Ionic liquids (ILs) have also been used as environmentally friendly extraction solvents in the DLLME method due to their advantages of good thermal stability, a low melting point, negligible vapor pressure, good miscibility with organic and aqueous solvents, and good extractabilities for various organic compounds^25,26^. ILs have been applied for extracting several organic compounds, such as pesticides and mycotoxins^27–33^. A study reported that IL-based DLLME has been used to extract pyrethroids in water^34^. In the IL-DLLME procedure, increasing the temperature and applying ultrasound energy to sample solutions have been used as the driving forces in the dispersion procedure^32,35–37^. The utilization of ultrasound energy could accelerate the mass transfer process and improve the extraction efficiency of ILs^38^. Moreover, the solubility of target analytes in ionic liquids can be optimized by changing the temperature. Therefore, combining UA with TCIL-DLLME (UA-TCIL-DLLME) is a very efficient strategy for improving the extraction efficiency. To date, there have been no reports about the application of the UA-TCIL-DLLME technique on the preconcentration of pyrethroid residues in herbal tea.

The goal of the present study was to establish an ultrasound-assisted temperature-controlled IL-DLLME (UA-TCIL-DLLME) method combined with HPLC-DAD for the analysis of five pyrethroids in herbal tea. Various factors affecting the enrichment of pyrethroids were investigated and optimized using single-factor experiments and response surface design (RSM). Finally, the established method was applied to detect pyrethroids in herbal tea samples.

## Results and discussion

### Optimization of the UETC-IL-DLLME technique

In determine the main parameters and their appropriate ranges, various factors, including the types and amount of the extractant, the kinds and volume of the dispersive solvent, the pH of the sample, the sonication time, the heating temperature, and the ionic strength, were investigated by single factor experiments with the other conditions remaining unchanged.

### Influence of kinds and volume of ionic liquid

**Figure 1:**
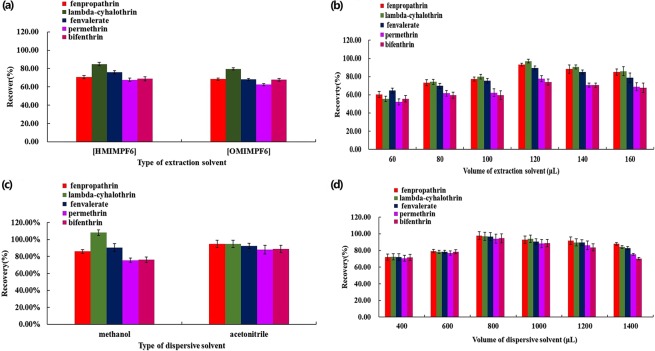
The effect of the type of extraction solvent (**a**), the amount of extraction solvent (**b**), the type of dispersive solvent (**c**) and the volume of dispersive solvent (**d**) on the extraction recoveries of five pyrethroids. Sample volume, 5 mL; added concentrations of the five pyrethroids: 0.1 mg L^−1^. Error bars representing the standard error (n = 5).

According to some liternature^39,40^, four ionic liquids, including in [HMIM][PF_6_], [OMIM][PF_6_], [BMIM][PF_6_] and [OMIM][PF_4_], were selected as extractants. In the present study, [OMIM][PF_4_] and [BMIM][PF_6_] were eliminated because only a samll amount of sediment phase was formed after centrifugation. Finally, [HMIM][PF_6_] was used as the extraction solvent because it had a higher extraction capability for the five target analytes than that of [OMIM][PF_6_] (Fig. 1a). Next, the effect of the [HMIM][PF_6_] amount on the extraction efficiency was tested by varying its volume in the range of 60–160 μL. The recoveries of the five analytes were gradually increased with increasing [HMIM][PF_6_] volume from 60 to 120 μL. When the volume of [HMIM][PF_6_] was increased beyond 120 μL, the extraction efficiency decreased (Fig. 1b). Therefore, 120 μL of [HMIM][PF_6_] was selected for subsequent experiments.

### Influence of kinds and volume of dispersive solvent

Two organic solvents, acetonitrile and methanol, which have appropriate miscibility in both the IL and aqueous phases, were selected for this study. As shown in Fig. 1c, the recoveries for the five targets were the highest when ACN was used as the dispersant. To evaluate the effect of dispersant volume, the volume of ACN was evaluated in the range of 400–1400 μL with a constant amount (120 μL) of [HMIM][PF_6_]. As shown in Fig. 1d, the best extraction efficiency was achieved when 800 μL of ACN was used.

### Influence of heating temperature and sonication time

**Figure 2:**
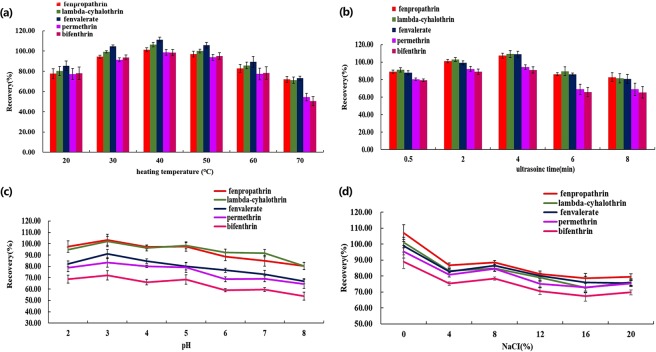
The effect of heating temperature (**a**), sonication time (**b**), pH (**c**) and NaCl (%) (**d**) on extraction recoveries of five pyrethroids. Extraction conditions: amount of [HMIM][PF6], 120 μL; volume of acetonitrile, 800 μL. Sample volumes, 5 mL; added concentrations of the five pyrethroids, 0.1 mg L^−1^. Error bars representing the standard error (n = 5).

Heating is an important factor in the extraction efficiency of the IL-DLLME process. Heating helps disperse ILs into the aqueous phase and influences the mass transfer rate of analytes. In the present study, the effects of temperature on the extraction efficiency were studied at 20–70 °C. The results exhibited that the highest recoveries were obtained at 40 °C (Fig. 2a). Sonication time can affect the formation of the finely dispersed mixture. The effect of the sonication time was evaluated in the range of 0.5–8 min. The extraction efficiency increased with increasing sonication time up to 4 min and then decreased from 4 min (Fig. 2b). Therefore, a sonication time of 4 min was chosen.

### Influence of pH

The sample pH is very important for the extraction of analytes. The effect of pH was investigated in the range of 2–8 by adding different amounts of HCl or NaOH into the herbal tea. The highest extraction recoveries were obtained at pH = 3.0, so a pH of 3.0 was used for subsequent experiments (Fig. 2c).

### Influence of salt addition

Ionic strength can improve the solubility of the target analytes in the extractant, thus enhancing the extraction efficiency. In the present study, different amounts of NaCl (from 0% to 8%) were added to the herbal tea samples to assess the influence of ionic strength. As seen in Fig. 2d, the extraction recoveries decreased when NaCl was added. Therefore, NaCl was not used in the IL-DLLME procedure.

### Optimization of extraction conditions by response surface methodology

After preliminary experiments, response surface methodology (RSM) based on the central composite design (CCD) was applied to select the optimal experimental conditions. Fifty experiments were carried out with randomly selected combinations of [HMIM][PF_6_] amount (A), acetonitrile volume (B), heating temperature (C), sonication time (D) and pH (E) using statistically designed experiments. The levels of each factor and high and low set points were established in an orthogonal design (Table 1). The lower and upper levels for each variable were selected after running preliminary experiments for each variable. The average recoveries of five pesticides as a function of A, B, C, D, and E were used as a response for the CCD design. The polynomial regression analysis was performed on the response values in the experiment, and a second-order polynomial equation was obtained:

$$\begin{array}{c}{\rm{R}}=0.94+0.024{\rm{A}}-0.012{\rm{B}}-0.021{\rm{C}}-0.044{\rm{D}}+0.015{\rm{E}}+0.009467{\rm{A}}{\rm{B}}\\ \,+\,0.11{\rm{A}}{\rm{C}}-0.009944{\rm{B}}{\rm{E}}-0.009216{\rm{C}}{\rm{D}}+0.022{\rm{D}}{\rm{E}}-0.043{{\rm{A}}}^{2}-0.032{{\rm{B}}}^{2}-0.017{{\rm{C}}}^{2}-0.010{{\rm{E}}}^{2}\end{array}$$

**Table 1:** The experimental range and levels of the variables in the CCD.

Variable	Parameter	Variable levels				
		−α (−2.378)	−1	0	1	+α (2.378)
*A*	Extraction solvent volume (μL)	110.5	116	120	124	129.5
*B*	Volume of CAN (μL)	700.1	758	800	842	900
*C*	Heating temperature (°C)	30.5	46	40	44	49.5
*D*	Ultrasonication time (min)	3	3.6	4	4.4	5
*E*	pH	1.8	2.5	3	3.5	4.2

ANOVA and regression analysis were used to evaluate the significance of the variables. As shown in Table S1, the coefficients of the five independent variables *A, B, C, D* and *E*, the four quadratic term coefficients of *A*^2^*, B*^2^*, C*^2^ and *E*^2^, and the four interactive cross-product coefficients *AB*, *AC, BE, and CD* with p-values <0.05, were significant for the extraction efficiencies of five pyrethroid pesticides. The model *F*-values of 40.54 indicated that the model was sufficient. The lack of fit (LOF) *P*-values (32.62) were not significant relative to the pure errors for the extraction efficiencies of 5 pyrethroids, which showed that the models fit well.

**Figure 3:**
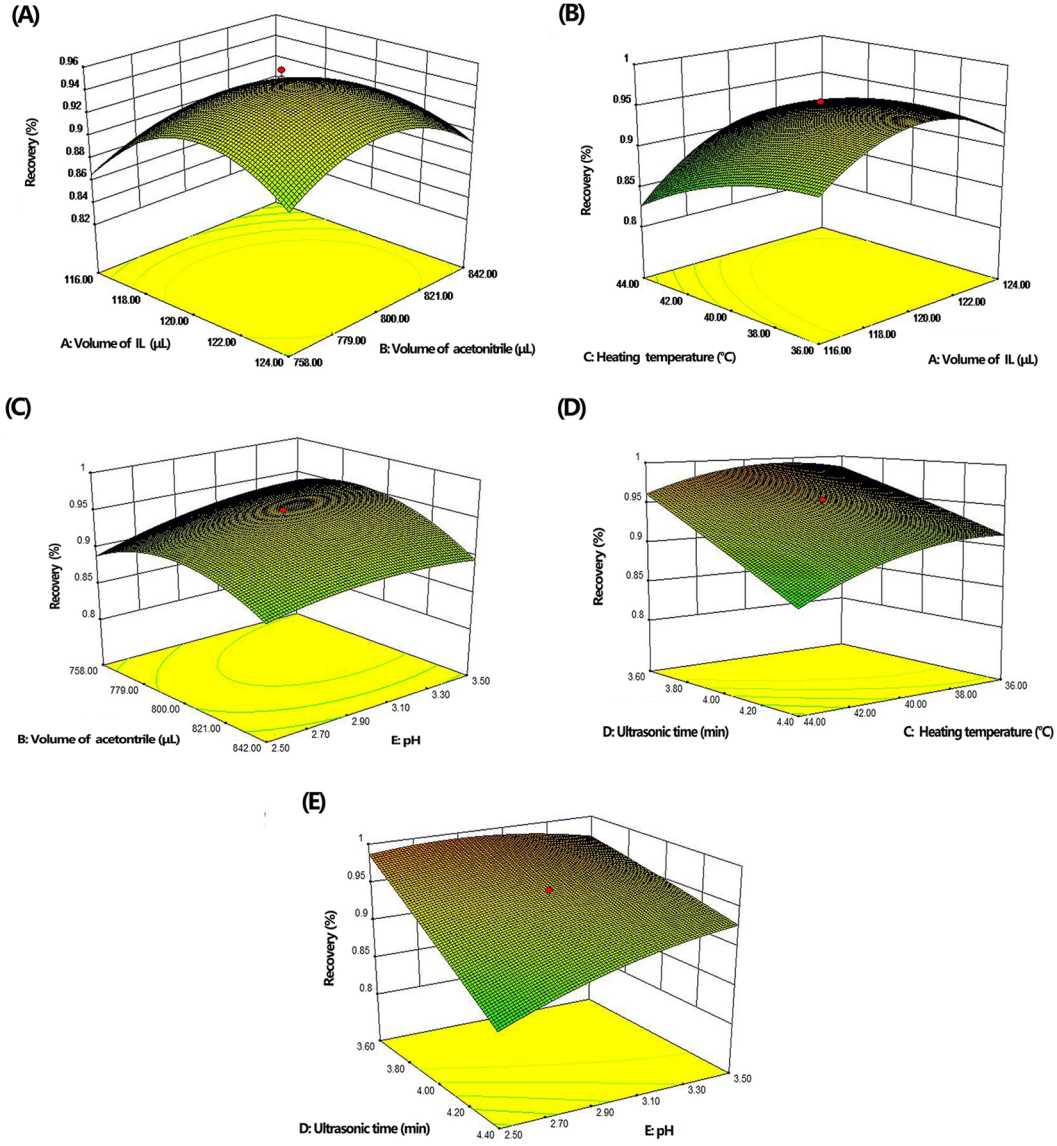
Response surface plots for the CCD: (**A**) amount of of [HMIM][PF6] and acetonitrile volume, (**B**) heating temperature and amount of [HMIM][PF6], (**C**) acetonitrile volume and pH, (**D**) sonication time and heating temperature, and (**E**) sonication time and pH.

From the designed models, three-dimensional surface and contour plots were obtained for each of the 5 pyrethroids, which graphically showed the relation between the five main factors and the response (Fig. 3). These plots were applied to determine the optimal conditions to achieve the highest extraction efficiencies for the 5 pyrethroids. Figure 3A shows the combined effect of the [HMIM][PF6] amount and acetonitrile volume. Figure 3B shows the response surface obtained by plotting the heating temperature versus the [HMIM][PF6] amount. Figure 3C shows the interaction between the acetonitrile volume and pH. Figure 3D shows the combined effect of sonication time and heating temperature. Figure 3E shows the response surface obtained by plotting sonication time versus pH. The optimal conditions predicated by RSM were 121 μL of [HMIM][PF6], 794 μL of the dispersive solvent, a heating temperature of 40 °C, 3.6 min of sonication time and a pH of 2.9.

### Analytical performance

The linearity and matrix effects were studied with calibration standards prepared in acetonitrile and in herbal tea with a linear range of 0.05–5 mg L^–1^. The results showed that the correlation coefficients (R) for the five pyrethroids ranged from 0.9993 to 1.0000 (Table 2). The following equation: ME = [(*A/B*) − 1] × 100%, is used to evaluate the matrix effect (ME); *A* and *B* are the slope ratios of calibration in the herbal tea matrix and in acetonitrile, respectively^41^. When ME is less than ±20%, the matrix effect is weak; The ME is between ±20% and ±50%, means that it is a medium matrix effect; The matrix effect is strong when the ME exceeds ±50%. As shown in Table 2, the MEs of five pesticides were below 7.94%, which confirmed that the herbal tea matrix did not interfere with the analyte signals. The limits of detection (LODs) calculated according to a signal-to-noise ratio of 3:1 (S/N = 3), and the LODs were in the range of 1.25–1.35 μg L^−1^ for the five pesticides. The limits of quantification (LOQs) were set to be the lowest addition concentration, and the LOQs of the five pesticides were all 0.005 mg L^−1^. In addition, the enrichment factor (EF), defined as the ratio between the concentration of the target in the sedimentary phase (*C*_*sed*_) and the initial concentration of the target in the aqueous phase (*C*_0_), was calculated using the following equation: EF = *C*_*sed*_*/C*_0_. The EF of the UETC-IL-DLLME system were ranged from 30 to 40, exhibiting sufficient enrichment ability for the five pesticides.

**Table 2:** Linear ranges, linearity, and matrix effects of the analytical method for the five pyrethroids.

Pesticides	Range (mg L^−1^)	Acetonitrile	Herbal tea A		Herbal tea B		LODs (µg L^−1^)	LOQs (µg L^−1^)
		Linearity (R^2^)	Linearity (R^2^)	Matrix Effect (%)	Linearity (R^2^)	Matrix Effect (%)		
Fenpropathrin	0.05 ∼ 5	1.0000	0.9999	3.21	0.9995	7.94	1.25	5
lambda-cyhalothrin	0.05 ∼ 5	0.9999	0.9999	0.33	0.9996	5.79	1.25	5
Fenvalerate	0.05 ∼ 5	0.9999	0.9999	1.05	0.9998	5.81	1.35	5
Permethrin	0.05 ∼ 5	0.9998	0.9999	1.23	0.9999	6.52	1.35	5
bifenthrin	0.05 ∼ 5	0.9994	0.9993	0.18	0.9995	8.24	1.35	5

### Application to real samples

**Figure 4:**
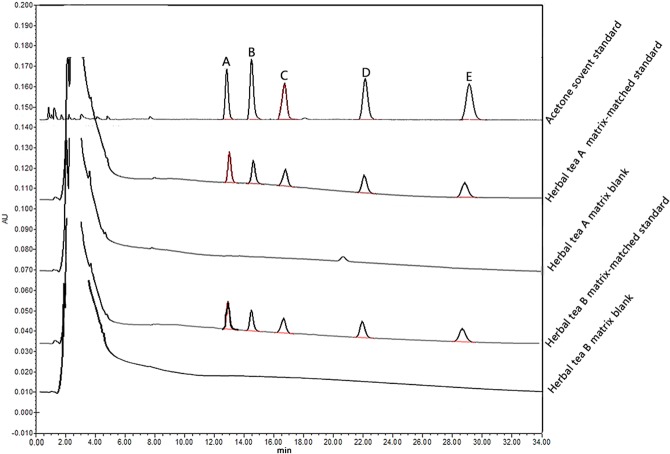
The typical HPLC chromatograms of the five pyrethroid pesticides in different matrix (**A**: Fenpropathrin; **B**: Lambda-cyhalothrin; **C**: Fenvalerate; **D**: Permethrin; **E**: Bifenthrin).

To validate the UETC-IL-DLLME method, recovery experiments were performed by spiking two herbal tea samples at four different concentrations (0.005, 0.05, 0.5 and 2.0 mg L^−1^) with the five pyrethroids. From the results as shown in Table 3, the average recoveries of the five pesticides in the two herbal tea samples were between 74.02% and 109.01%, with RSDs of 3.41–9.04%. Thus, UETC-IL-DLLME could be applied for the analysis of herbal tea samples with weak matrix effects. The established method was used to detect the five targets in 10 batches of herbal tea samples, which were purchased from local markets in Guangzhou, China, in 2018. No pesticide residues were detected (less than LOD) in any herbal tea sample. The typical chromatograms are shown in Fig. 4.

**Table 3:** Mean recoveries (%) and relative standard deviations (%) of five pyrethroid pesticides in spiked herbal tea samples after UETC-IL-DLLME-HPLC-DAD.

Pesticides	Spiked level (mg L^−1^)	Herbal tea A		Herbal tea B	
		Mean recoveries (%)	RSD (%)	Mean recoveries (%)	RSD (%)
Fenpropathrin	0.005	74.02	5.79	103.05	7.68
	0.05	83.22	3.64	95.55	8.49
	0.5	95.61	9.04	100.16	7.15
	2	97.18	5.34	91.56	4.42
lambda-cyhalothrin	0.005	103.05	7.68	99.84	7.35
	0.05	98.29	8.92	106.16	3.41
	0.5	102.12	8.82	109.01	8.74
	2	101.18	4.92	98.03	4.76
Fenvalerate	0.005	99.84	7.35	87.29	6.21
	0.05	74.96	5.22	86.76	6.37
	0.5	86.38	8.76	90.74	8.05
	2	87.18	5.19	83.57	4.71
Permethrin	0.005	87.29	6.21	86.01	7.78
	0.05	84.26	8.74	98.43	6.09
	0.5	97.90	8.72	102.89	8.11
	2	101.73	5.69	95.62	4.86
bifenthrin	0.005	96.41	7.52	85.98	5.11
	0.05	85.81	5.07	96.49	6.80
	0.5	108.75	7.71	107.46	8.31
	2	108.97	7.55	108.51	4.94

## Material and methods

### Chemicals and standards

Standards of fenpropathrin (98.36%), permethrin (99.37%), bifenthrin (99.71%) and fenvalerate (98.37%) were purchased from Shanghai Anpel Scientific Instrument Corporation (Shanghai, China), and lambda-cyhalothrin (98.5%) was purchased from Beijing Century Trade Co., Ltd. (Beijing, China). The ionic liquids, 1-octyl-3-methylimidazolium hexafluorophosphate ([OMIM]PF6), 1-hexyl-3-methylimidazolium hexafluorophosphate ([HMIM]PF6), (1-butyl-3-methylimidazole hexafluorophosphate) ([BMIM]PF6), and (1-octyl-3-methylimidazole tetrafluorophosphate) ([OMIM]BF4), were purchased from Beijing Century Trade Co., Ltd (Beijing, China). The chemical structures of the five pyrethroids and four ionic liquids are shown in Figures S1 and S2, respectively.

Sodium chloride (NaCl), hydrochloric acid (HCl) and sodium hydroxide (NaOH) were obtained from Guangzhou Qianye Instrument Co., Ltd (Guangzhou, China). Acetonitrile (ACN, HPLC grade) and methanol (MeOH, HPLC grade) were purchased from Shanghai Anpel Scientific Instrument Corporation (Shanghai, China). Ultrapure water (UNIQUE-R20 purification system, China) was used in our study.

### Instruments and equipment

A high-performance liquid chromatography system (Waters 12695, American), which consisted of a quaternary pump and a DAD detector, was used to analyze the five pyrethroid pesticides. Separation was performed with a ZORBAX Eclipse C_18_ column (5 μm, 4.6 × 150 mm, Waters) at a temperature of 30 °C. Acetonitrile-water (78:22, v/v) was used as the mobile phase at a flow rate of 1.0 mL/min. The detection wavelength was set at 205 nm, and the injection volume was 10 μL.

### UETC-IL-DLLME procedure

Five milliliters of filtered herbal tea (pH 2.9) was transferred into a 15 mL conical centrifuge tube. A mixture of 121 μL of [HMIM]PF6 (extraction solvent) and 794 μL of acetonitrile (dispersant) was quickly injected into the herbal tea, and a cloudy solution was formed. The tube was placed into an ultrasonic water bath at 40 °C for 3.6 min. Then, the tube was cooled to obtain the cloudy state again. The mixture was centrifuged for 6 min at 3800 rpm, and the supernatant was removed. Approximately 130 ± 5 μL of sedimented IL was collected at the bottom of the centrifuge tube. Fifty microliters of sedimented IL was dissolved in 50 μL of acetonitrile and filtered through 0.22 μm cellulose membrane filters. Ten microliters of the resulting solution was then injected into the HPLC system for analysis.

### Experimental design and data analysis

Response surface methodology (RSM) based on a central composite design (CCD) was applied to optimize the extraction conditions affecting the efficiency of the UETC-IL-DLLME process. First, a screening study was used to determine the main parameters and their appropriate ranges. Five independent variables were chosen as follows: A, [HMIM][PF_6_] amount; B, acetonitrile volume; C, heating temperature; D, sonication time; and E, pH. Five levels of each variable were studied and coded as −α, −1, 0, +1, and −α, with α = 2.378 (Table 1). The CCD contained fifty experimental trials that had eight repeated trials of the central points. The fifty trials, including eight factorial points, six axial points and eight replicated center points, were proposed by Design Expert software 8.0.5 (Minneapolis, MN, USA), which was applied for the design, analysis and optimization of the experiments.

## Supplementary information

Supplementary information.
