# Supplementary material for: Determination of pyrethroid residues in herbal tea using temperature-controlled ionic liquid dispersive liquid-liquid microextraction by high performance liquid chromatography
Source: Sci Rep. 2020 Mar 13;10:4709. doi: 10.1038/s41598-020-61755-z (PMC7070011; doi:10.1038/s41598-020-61755-z)
Supplement: Supplementary file 1 — Supplementary information. [file 41598_2020_61755_MOESM1_ESM.pdf]

Supporting Information

Determination of pyrethroids residues in herbal tea using temperature-controlled ionic liquid dispersive liquid-liquid microextraction by high performance liquid chromatography

Rui Zhang[†], Zhenchao Tan[†], Junlong Zhao[†], Yan Wen[†], Shuai Fan[†] and Chenglan Liu[†]*

†Key Laboratory of Natural Pesticide and Chemical Biology, Ministry of Agriculture& Key Laboratory of Bio-Pesticide Innovation and Application of Guangdong Province, South China Agricultural University, 483 Wushan Road, Guangzhou, 510642, China

[*] Correspondence: liuchenglan@scau.edu.cn; Tel.: +86-20-85284925

Table S1

Analysis of variance (ANOVA) for the response surface quadratic model

Source	Sum of squares	Degree of freedom	Mean square	*F*-value	*p*-value	Prob.＞F
Model	0.35	20	0.017	40.54	< 0.0001	significant
A-[HMIM][PF6] amount (mL)	0.026	1	0.026	60.85	< 0.0001	
B-volume of acetonitrile (mL)	6.08E-03	1	6.08E-03	14.26	0.0014	
C-heating temperature (℃)	0.019	1	0.019	45.65	< 0.0001	
D-ultrasonic time (min)	0.085	1	0.085	199.97	< 0.0001	
E-pH	9.78E-03	1	9.78E-03	22.91	0.0001	
AB	2.87E-03	1	2.87E-03	6.72	0.0217	
AC	3.97E-03	1	3.97E-03	9.3	0.0079	
AD	2.02E-03	1	2.02E-03	4.74	0.0508	
AE	1.02E-03	1	1.02E-03	2.38	0.1597	
BC	2.27E-04	1	2.27E-04	0.53	0.5007	
BD	1.51E-05	1	1.51E-05	0.035	0.8617	
BE	3.10E-03	1	3.10E-03	7.27	0.0174	
CD	2.72E-03	1	2.72E-03	6.37	0.0251	
CE	6.68E-06	1	6.68E-06	0.016	0.9076	
DE	0.016	1	0.016	36.55	< 0.0001	
A^2	0.11	1	0.11	263.08	< 0.0001	
B^2	0.065	1	0.065	153.25	< 0.0001	
C^2	0.019	1	0.019	44.62	< 0.0001	
D^2	9.75E-05	1	9.75E-05	0.23	0.8462	
E^2	8.15E-03	1	8.15E-03	19.09	0.0018	
Residual	0.012	29	4.27E-04			
Lack of Fit	0.012	22	5.57E-04	32.62	0.1064	
Pure Error	1.59E-03	7	2.27E-04			
Cor Total	0.35	49				

R^2: 0.9591; Adj. R^2: 0.9308; CV=2.6%

Figure S1

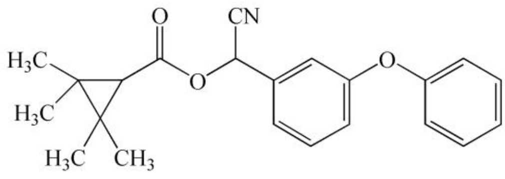

fenpropathrin

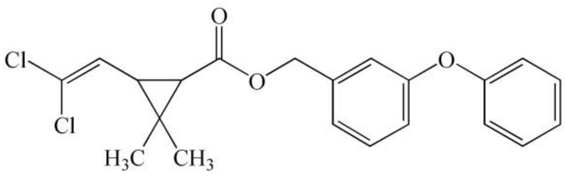

permethrin

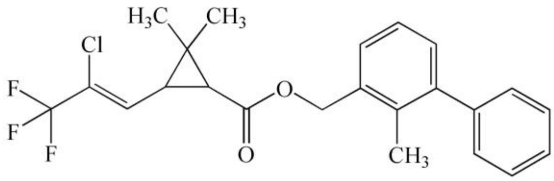

bifenthrin

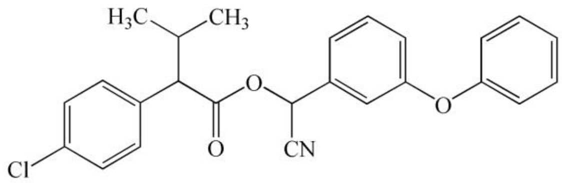

fenvalerate

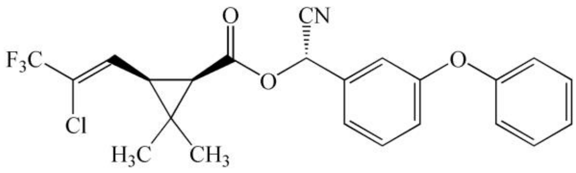

lambda-cyhalothrin

Figure S1 The chemical structures of five pyrethroids

Figure S2

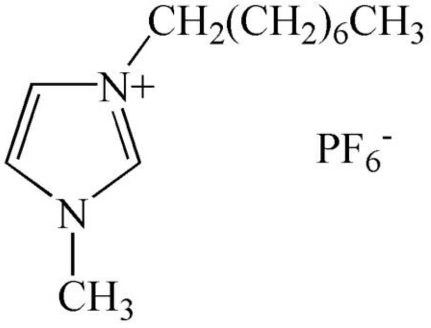

$[OMIM]PF_6$

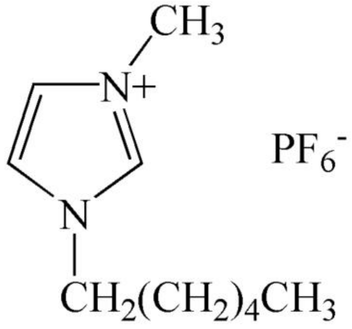

$[HMIM]PF_6$

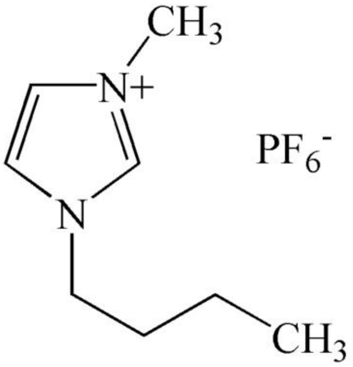

$[BMIM]PF_6$

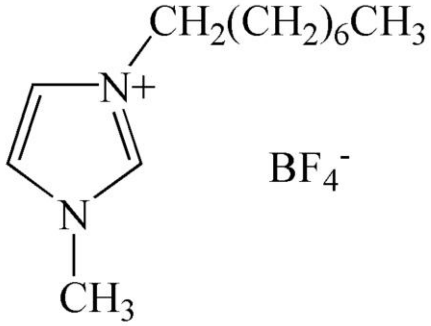

$[OMIM]BF_4$

Figure S2 The chemical structures of four ionic liquids.
